# PReS-FINAL-2346: Hypomorphic RAG deficiencies: phenotypic variability and usefulness of TREC/KREC as diagnostic biomarkers

**DOI:** 10.1186/1546-0096-11-S2-P336

**Published:** 2013-12-05

**Authors:** C Schuetz, U Pannicke, EM Jacobsen, M Honig, T Niehues, K Schwarz, AS Schulz

**Affiliations:** 1 Pediatrics and Adolescent Medicine, Ulm University, Ulm, Germany; 2 Institute for Transfusion Medicine, ULM UNIVERSITY, Ulm, Ulm, Germany; 3 Center for Pediatric and Adolescent Health, HELIOS Klinikum Krefeld, Krefeld, Ulm, Germany; 4 Institute for Clinical Transfusion Medicine and Immunogenetics, Ulm University, Ulm, Germany

## Introduction

An increasing number of patients with combined immunodeficiencies have been found to carry hypomorphic variants of genes that otherwise cause severe combined immunodeficiency (SCID). These patients not only present with recurrent and sometimes life-threatening infections, but also with immunodysregulatory symptoms such as autoimmune cytopenias and granulomas, and are a diagnostic challenge.

## Objectives

The objective of this study was to determine whether TRECs and KRECs are useful in identifying patients beyond infancy with combined T- and B-cell deficiencies who are otherwise difficult to diagnose due to late-onset, heterogeneous clinical phenotypes and variable numbers and functions of T and B cells.

## Methods

Patients with combined immunodeficiency due to *hRAG *mutations (n = 6), with DOCK8 deficiency (n = 3) and with classical SCID due to RAG1, RAG2, ARTEMIS and IL2RG defects were treated at the University Hospital Ulm, Germany. Four of the patients with hRAG deficiencies presented with granulomatous lesions, one with vitiligo, three patients had autoimmune cytopenias. The earliest available samples of MNCs were analyzed (cells cryopreserved at the median age of 10 years, range 6-17 years) for patients with hRAG and DOCK-8 deficiencies.

## Results

Immunophenotyping of hRAG patients' peripheral MNCs showed reduced, but variable numbers of T and B cells. Thymic derived naïve CD3^+^CD45RA^+ ^T cells were < 30% in all patients (range 1-27%). Residual T-cell function (proliferation assays) and B-cell function (antibody titres following vaccination, data not shown) were detectable, but abnormal in all hRAG patients. T-cell repertoires were diverse in 5 patients, and restricted in patients 1 and 4. Even when detectable, TREC and KREC amounts in hRAG patients were at least 32-fold reduced compared to healthy controls

## Conclusion

Measurement of TREC and KREC levels is a fast and easily performed tool for the quantification of thymic output and B-cell maturation respectively. We envisage that these biomarkers may serve as valuable complementary parameters for the initial immunological work-up when a diagnosis of CID is being considered. Timely clinical suspicion paired with abnormal TREC and KREC levels might facilitate earlier referral of this group of patients with atypical and late presentation of CID to the pediatric immunologist and possibly to treatment by HCT.

## Disclosure of interest

None declared.

